# Downregulation of Three Immune-Specific Core Genes and the Regulatory Pathways in Children and Adult Friedreich's Ataxia: A Comprehensive Analysis Based on Microarray

**DOI:** 10.3389/fneur.2021.816393

**Published:** 2022-02-14

**Authors:** Lichun Liu, Yongxing Lai, Zhidong Zhan, Qingxian Fu, Yuelian Jiang

**Affiliations:** ^1^Department of Pharmacy, Fujian Children's Hospital, Fuzhou, China; ^2^Department of Geriatric Medicine, Fujian Provincial Hospital, Fuzhou, China; ^3^Department of Pediatric Intensive Care Unit, Fujian Children's Hospital, Fuzhou, China; ^4^Department of Pediatric Endocrinology, Fujian Children's Hospital, Fuzhou, China

**Keywords:** Friedreich's ataxia, biomarker, hub genes, bioinformatics, RNA regulatory pathways

## Abstract

**Background:**

Friedreich's ataxia (FRDA) is a familial hereditary disorder that lacks available therapy. Therefore, the identification of novel biomarkers and key mechanisms related to FRDA progression is urgently required.

**Methods:**

We identified the up-regulated and down-regulated differentially expressed genes (DEGs) in children and adult FRDA from the GSE11204 dataset and intersected them to determine the co-expressed DEGs (co-DEGs). Enrichment analysis was conducted and a protein-protein interaction (PPI) network was constructed to identify key pathways and hub genes. The potential diagnostic biomarkers were validated using the GSE30933 dataset. Cytoscape was applied to construct interaction and competitive endogenous RNA (ceRNA) networks.

**Results:**

Gene Set Enrichment Analysis (GSEA) indicated that the genes in both the child and adult samples were primarily enriched in their immune-related functions. We identified 88 co-DEGs between child and adult FRDA samples. Gene Ontology (GO), Kyoto Encyclopedia of Genes and Genomes (KEGG), and Reactome enrichment analysis suggested that these co-DEGs were primarily enriched in immune response, inflammatory reaction, and necroptosis. Immune infiltration analysis showed remarkable differences in the proportions of immune cell subtype between FRDA and healthy samples. In addition, ten core genes and one gene cluster module were screened out based on the PPI network. We verified eight immune-specific core genes using a validation dataset and found CD28, FAS, and ITIF5 have high diagnostic significance in FRDA. Finally, NEAT1-hsa-miR-24-3p-CD28 was identified as a key regulatory pathway of child and adult FRDA.

**Conclusions:**

Downregulation of three immune-specific hub genes, CD28, FAS, and IFIT5, may be associated with the progression of child and adult FRDA. Furthermore, NEAT1-hsa-miR-24-3p-CD28 may be the potential RNA regulatory pathway related to the pathogenesis of child and adult FRDA.

## Introduction

Friedreich's ataxia (FRDA) is a familial hereditary disorder involving the spinal cord and cerebellum, which is mainly caused by repeat amplification of homozygous guanine–adenine–adenine (GAA) triplet located in the frataxin gene (1). Such repeat amplification and mutation eventually lead to a decrease in the expression level of functional Frataxin. Frataxin deficiency can promote the activation of oxidative stress and ferroptosis, resulting in mitochondrial dysregulation (2, 3). Children often manifest with initial symptoms including disturbance of balance and progressive ataxia. With the progress of disease and age, patients may gradually develop dysarthria and loss of tendon reflex, and in many patients this is accompanied by myocardial injury and diabetes (4). At present, no effective therapies have been proven to prevent FRDA progression, most of which are symptomatic treatment (5). Therefore, further elucidating the underlying pathogenesis and developing more valid treatment strategies is an urgent demand. Presently, some serum biomarkers have been reported as potential key signatures in the pathogenesis of FRDA. For example, the levels of neurofilament light chain and heavy chain are enhanced significantly in Friedreich's ataxia patients and decrease with age (6, 7). In addition, serum hsTnT, NT-proBNP, and miRNAs have also been demonstrated to be associated with the progression of cardiomyopathy in adult FRDA (8, 9). However, the effectiveness of these biomarkers has either not been validated in prospective cohorts, or the clinical correlation between them is barely understood. Moreover, these biomarkers have not yet been used in clinical diagnosis of FRDA. Therefore, identifying additional biomarkers may provide critical insights into the diagnosis and treatment of FRDA.

Currently, bioinformatics analysis has been extensively applied in a variety of diseases, including cancer, cardiac disease, and neurodegenerative disease, to identify key biomarkers closely related to the prognosis of the disease (10–12). Additionally, competitive endogenous RNA (ceRNA) networks will help to clarify the novel mechanism of transcriptional regulatory networks in advancing disease progression (13). Although recent studies have concentrated on FRDA-induced transcriptome changes, only a few studies have explored the association between differentially expressed genes (DEGs) in children and adult FRDA.

In this study, we identified co-expressed differentially expressed genes (co-DEGs) by intersecting the up-regulated and down-regulated DEGs in child and adult FRDA samples (GSE11204). Next, we performed various enrichment analysis and constructed a PPI network to ascertain the key pathways and hub genes related to the progression of FRDA in children and adults. In addition, we predicted target miRNAs of hub genes and validated the diagnostic significance of selected hub genes using the GSE30933 dataset, Finally, we constructed FRDA-related ceRNA networks based on mRNAs-miRNAs-long noncoding RNAs (lncRNAs) interactions. Our study provides a novel perspective for revealing the pathophysiological mechanism of FRDA progression at the transcriptome level and investigates potential targets for the diagnosis and treatment of FRDA in children and adults.

## Materials

### Microarray Data Acquisition

All microarray datasets were downloaded from Gene Expression Omnibus (GEO) (www.ncbi.nlm.nih.gov/geo/) (14). GSE11204 dataset (GPL887 platform) (15), including whole gene expression profiles of peripheral blood from 10 healthy children, 28 children with FRDA, 15 healthy adults, and 14 adults with FRDA, were selected as the test set. The GSE30933 dataset (GPL6255 platform) (16), which included whole gene expression profiles of peripheral blood from 40 healthy and 34 FRDA samples, was selected as the validation set.

### Data Processing

The whole gene expression profiles obtained from the GEO database have been pre-processed and normalized by the robust multi-array average (RMA) method according to the “affy” package (http://www.bioconductor.org/packages/release/bioc/html/affy.html) (version 1.70.0) of R software. The “limma” package (http://www.bioconductor.org/packages/release/bioc/html/limma.html) (version 3.48.1) was performed to analyze the differentially expressed genes (DEGs). Original *p*-values were adjusted by the Benjamini-Hochberg method, and the fold-changes (FC) were calculated based on the false detection rate (FDR) procedure. Genes expression values of |log2 FC| > 1 and *p* < 0.05 were considered to be statistically significant. In order to visualize the identified DEGs, the R packages of “ggpubr” and “pheatmap” were conducted to make the volcano plots and heatmaps, respectively. The online tool Draw Venn Diagram (http://bioinformatics.psb.ugent.be/webtools/Venn/) was conducted to generate Venn diagrams of co-DEGs.

### Enrichment Analysis

To identify the distribution trend of overall genes between the FRDA and the control groups, the “clusterProfiler” (http://www.bioconductor.org/packages/release/bioc/html/clusterProfiler.html) (version 4.0.2) and “msigdbr” (https://cran.r-project.org/web/packages/msigdbr/index.html) (version 7.4.1) packages were conducted to make Gene Set Enrichment Analysis (GSEA) enrichment analysis. In brief, the gene symbols with corresponding FC were imported, and the c5: GO:BP gene sets (c5.go.bp.v7.4.symbols) were then applied for functional enrichment analysis. Gene sets with *p* < 0.05, Q <0.25, and | normalized enrichment score (NES)| > 1.5 were defined as significantly enriched gene sets.

Next, Gene Ontology (GO) enrichment analysis of co-DEGs was carried out based on the Database for Annotation, Visualization and Integrated Discovery (DAVID) (https://david.ncifcrf.gov/summary.jsp) (17). The biological process (BP), cell composition (CC), and molecular function (MF) of co-DEGs were then identified. Enriched GO terms (BP) with FDR <0.05 were defined as significant and visualized using the “GOplot” package (https://wencke.github.io/) (version 1.0.2).

In addition, Kyoto Encyclopedia of Genes and Genomes (KEGG) and Reactome enrichment analysis of co-DEGs were conducted with the “clusterProfile” package (18). A value of adjusted *p* < 0.05 was considered as significantly enriched functions and pathways. The top 5 KEGG and Reactome enrichment pathways were exhibited in a bubble plot.

### Protein-Protein Interaction Network Analysis

The PPI network of co-DEGs was established using the online database STRING (https://string-db.org/) based on the Screening criteria: combined score > 0.4 (19). Afterward, the protein-protein interaction information was imported into the Cytoscape software (3.8.2) to realize the visualization of the PPI network. Then, the Minimal Common Oncology Data Elements (MCODE) plugin was applied for identifying key gene clusters with the default parameters. CytoHubba plugin was carried out to screen out hub genes based on the PPI network (20). The first 20 hub genes were calculated using the five algorithms: Degree, Stress, Maximum Neighborhood Component (MNC), Closeness, and Radiality (21, 22). Finally, a total of 10 hub genes were screened out by intersecting all the results.

### Gene-miRNA Analysis

Gene-miRNA interactions were identified using the online database miRWalk 3.0 (http://mirwalk.umm.uni-heidelberg.de/) (23). The target miRNAs of selected genes were predicted using the miRWalk and miRDB databases with the default parameters (*p* < 0.05, seed sequence lengths more than 7 mer, the target gene-binding regions: 3′ UTR). The miRNAs were selected by intersecting all the results. Finally, mRNA-miRNA interaction network was constructed and visualized using Cytoscape software.

### Functional Analysis of Target miRNAs

All the predicted target miRNAs were uploaded to the Funrich software (3.1.2), the molecular functions and biological pathways of target miRNAs were then identified. *p* < 0.05 was defined as markedly enriched functions and pathways.

### Construction of ceRNA Networks

The target lncRNAs interacting with the selected miRNAs were predicted using the online database StarBase 3.0 (24). We selected the lncRNAs with most of the cross-linked miRNAs as our predicted lncRNAs according to the following screening criteria: mammalian, human h19 genome, CLIP-Data more than 5, and with or without degradome data. CeRNA networks based on the mRNAs-miRNAs-lncRNAs interactions were constructed and visualized using Cytoscape software.

### Immune Infiltration Analysis

CIBERSORT is an analytic algorithm based on the gene expression profiles of 547 genes. The CIBERSORT could calculate the compositions of different immune cell subtypes using the deconvolution precisely on the algorithm, thus exhibiting the signature of each immune cell subtype (25). The gene expression profiles of all genes or co-DEGs including Control and FRDA samples were imported to perform the immune infiltration analysis using the CIBERSORT algorithm through R software. Student's *T*-tests were conducted to analyze the difference in the proportion of each immune cell subtype between Control and FRDA samples. A value of *p* < 0.05 was considered statistically significant. Finally, the “ggplot” (https://cran.r-project.org/src/contrib/Archive/ggplot/) (version 0.4.2) and “ggplot2” (http://had.co.nz/ggplot2/) (version 3.3.5) packages were performed to visualize the results.

### Statistics Analysis

The “ggpubr” package (https://cran.r-project.org/web/packages/ggpubr/index.html) version 0.4.0) was applied to perform statistical analyses, the “ggplot2” and “ggplot” packages were conducted to draw boxplots and bar plots, respectively. The “GOplot” package was used to draw a chord plot. Student's *t*-test was performed to analyze the differences between the two groups.

## Results

### Identification of DEGs

**Figure 1:**
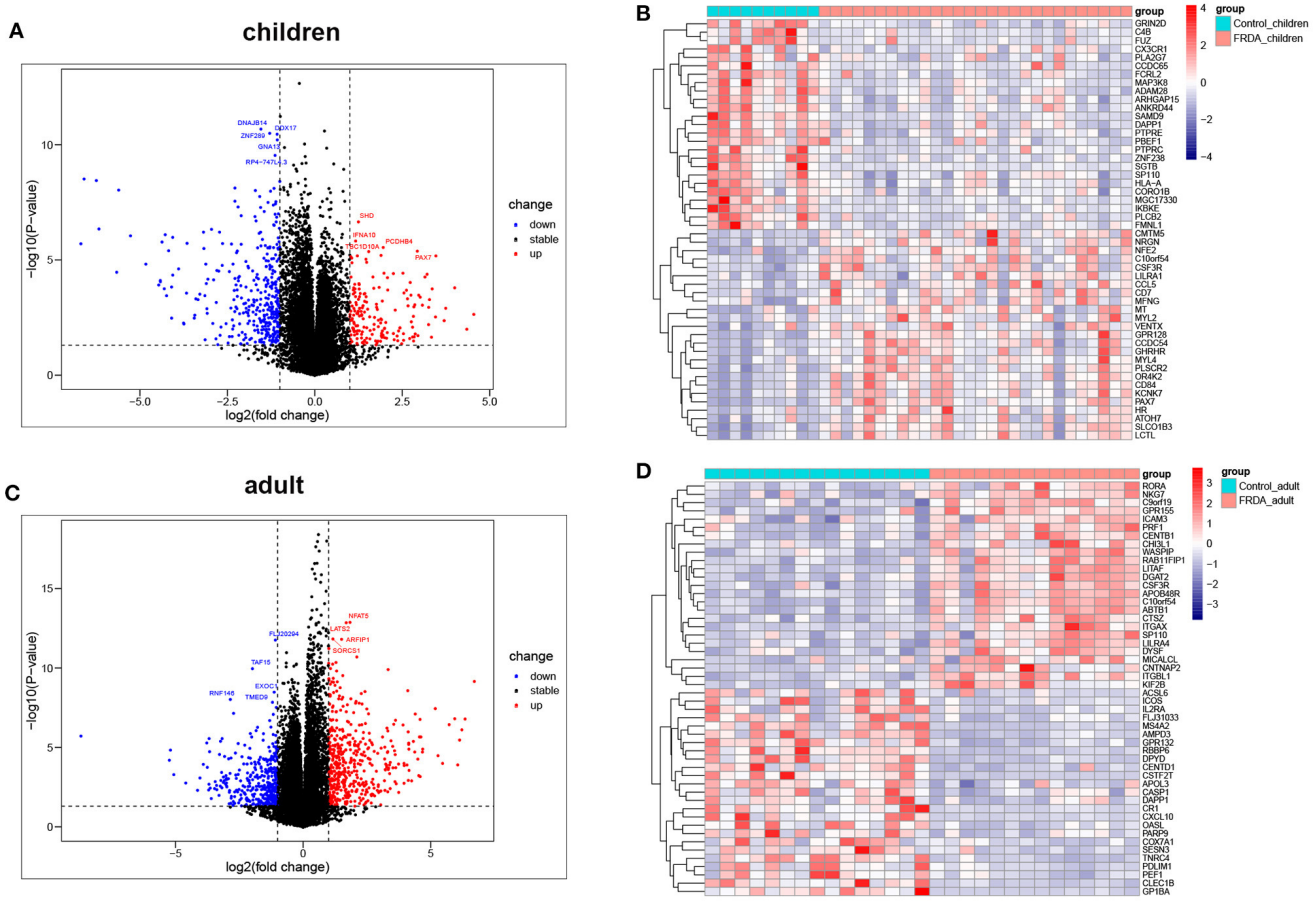
Identification of DEGs between Control and FRDA samples. **(A)** The volcano plot of the genes in children samples. **(B)** Representative heatmap of DEGs in children samples. The top 25 up-regulated and down-regulated DEGs are shown, respectively. **(C)** The volcano plot of the genes in adult samples. **(D)** Representative heatmap of DEGs in adult samples. The top 25 up-regulated and down-regulated DEGs are exhibited, respectively. Red points: up-regulated DEGs, blue points: down-regulated DEGs, gray points: no changed genes; Red rectangles: high expression, blue rectangles: low expression.

The dataset GSE11204, which included 10 healthy children, 28 children with FRDA, 15 healthy adults, and 14 adults with FRDA, were selected to analyze and identify the DEGs. We screened out a total of 530 (177 up-regulated and 353 down-regulated) DEGs in child samples and a total of 857 (483 up-regulated and 374 down-regulated) DEGs in adult samples. The volcano plots exhibited the number of DEGs identified from child and adult samples, respectively. The heatmaps displayed the expression of the top 25 up-regulated and down-regulated DEGs in child and adult samples, respectively (Figures 1A–D). The expression levels of all DEGs in child and adult samples were also visualized in heatmaps (Supplementary Figures 1A,B).

### GSEA and Immune Infiltration Analysis

**Figure 2:**
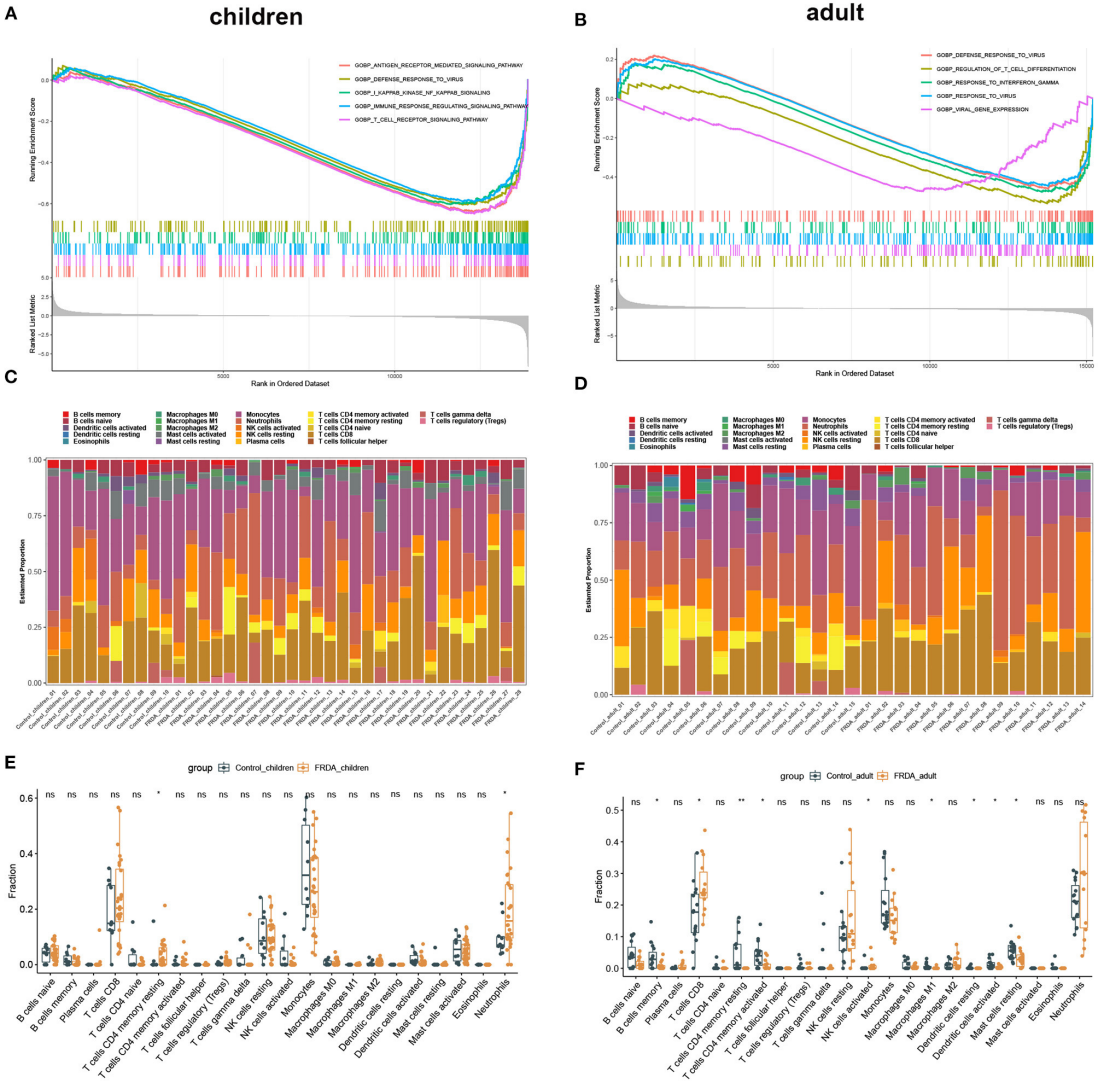
GSEA and Immune infiltration analysis in children and adult samples. **(A,B)** GSEA between Control and FRDA group in children **(A)** and adult **(B)**. The top 5 gene sets were exhibited. **(C,D)** The landscape of all 22 types of immune cell components in children **(C)** and adults **(D)**. **(E,F)** Boxplots of the differentially infiltrated immune cells between Control and FRDA group in children **(E)** and adult (F). *p < 0.05, **p < 0.01 when compared with the Control group.

In order to clarify the biological process and the immune cell subtype involved in the child and adult samples, respectively, we firstly performed the GSEA and found the genes in children were significantly enriched in antigen-receptor mediated signaling pathway, defense response to virus, NF-κB signaling, regulation of immune response signaling pathways, and T cell receptor signaling pathways (Figure 2A). In adults, the enriched gene sets were mainly involved in defense response to virus, regulation of T cell differentiation, response to interferon-gamma, response to virus, and viral gene expression (Figure 2B). Subsequently, we applied immune infiltration analysis and found the proportions of immune cell subtype distinct between groups (Figures 2C,D). Compared with the Control_children group, the FRDA_children group contained a greater number of resting memory CD4+ T cells and Neutrophils (Figure 2E). In addition, the FRDA_adult group markedly elevated the number of CD8+ T cells and activated NK cells, whereas the number of memory B cells, resting memory CD4+ T cells, activated memory CD4+ T cells, M1 Macrophages, resting Dendritic cells, activated Dendritic cells, and resting Mast cells decreased when compared with the Control_adult group (Figure 2F).

### Identification of Co-DEGs and Functional Enrichment Analysis

**Figure 3:**
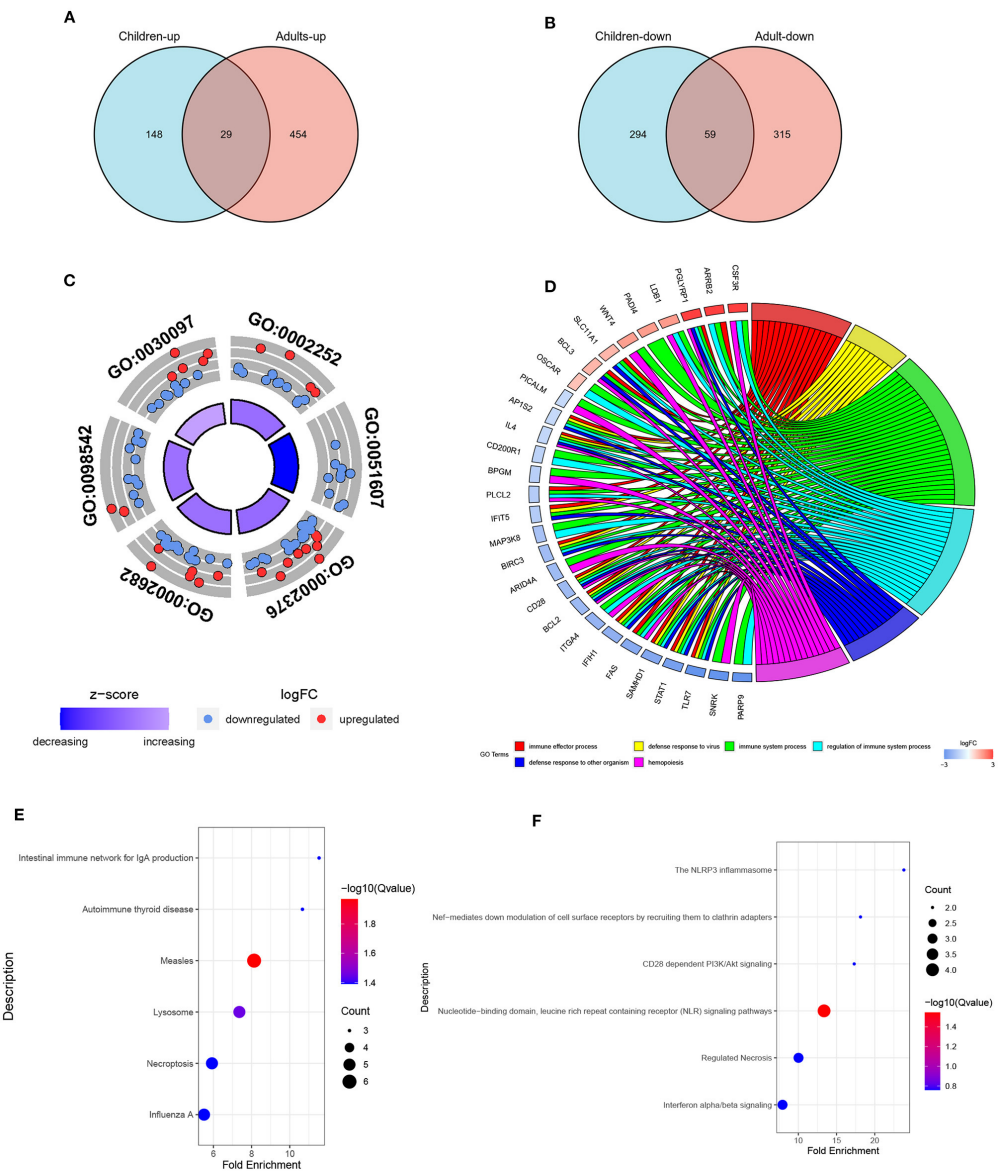
The intersections of DEGs between groups and functional enrichment analysis of co-DEGs. **(A,B)** Venn diagram of common up-regulated and down-regulated DEGs between children **(A)** and adults **(B)**. **(C)** Top 6 GO terms (BP) of the 88 co-DEGs with the DAVID analysis. **(D)** Representative chord plot of the top 6 GO terms (BP) of co-DEGs. **(E,F)** Representative bubble plot of the top 5 enriched KEGG (E) and Reactome **(F)** pathways of co-DEGs.

The DEGs from the child and adult datasets were intersected and eventually identified 29 co-up-regulated and 59 co-down-regulated DEGs (Figures 3A,B). Therefore, we screened out a total of 88 co-DEGs. To further explore the enrichment pathways involved in these co-DEGs, we firstly performed GO enrichment analysis using the DAVID website. These co-DEGs were primarily involved in the biological processes including the immune effector process, defense response to virus, immune system process, defense response to other organisms, and hemopoiesis (Table 1 and Figures 3C,D). In addition, KEGG pathway enrichment analysis suggested that these co-DEGs were mainly involved in the intestinal immune network for IgA production, autoimmune thyroid disease, measles, lysosome, necroptosis, and influenza A (Figure 3E). Reactome enrichment analysis indicated that these co-DEGs were primarily enriched in the immune system, inflammatory reaction, necrosis, and signal transduction (Figure 3F).

**Table 1:** Top 6 GO terms (BP) of the 88 co-DEGs with the DAVID analysis.

**ID**	**Term**	**Count**	**Genes**	**Fold enrichment**	**FDR**
GO:0002252	immune effector process	16	STAT1, SLC11A1, PLCL2, IFIT5, ARRB2, SAMHD1, IFIH1, IL4, BCL3, AP1S2, CD28, BCL2, FAS, TLR7, PGLYRP1, BIRC3	4.82789	6.64E-04
GO:0051607	defense response to virus	10	IFIH1, IL4, STAT1, BCL2, AP1S2, IFIT5, CD28, TLR7, SAMHD1, BIRC3	9.77700	6.64E-04
GO:0002376	immune system process	29	CSF3R, LDB1, IFIT5, ARID4A, ARRB2, SAMHD1, IFIH1, AP1S2, MAP3K8, PGLYRP1, WNT4, ITGA4, STAT1, SLC11A1, PLCL2, BPGM, PARP9, OSCAR, IL4, SNRK, CD200R1, BCL3, BCL2, CD28, FAS, TLR7, PADI4, BIRC3, PICALM	2.63404	6.64E-04
GO:0002682	regulation of immune system process	21	CSF3R, ITGA4, LDB1, STAT1, SLC11A1, PLCL2, ARRB2, SAMHD1, PARP9, OSCAR, IFIH1, IL4, CD200R1, AP1S2, CD28, BCL2, FAS, MAP3K8, TLR7, PGLYRP1, BIRC3	3.41202	6.64E-04
GO:0098542	defense response to other organism	13	STAT1, SLC11A1, IFIT5, SAMHD1, IFIH1, IL4, BCL3, AP1S2, CD28, BCL2, TLR7, PGLYRP1, BIRC3	5.89193	6.64E-04
GO:0030097	hemopoiesis	15	CSF3R, ITGA4, LDB1, PLCL2, ARID4A, BPGM, IL4, SNRK, BCL3, CD28, BCL2, FAS, PGLYRP1, PICALM, WNT4	4.54452	0.00131

**Figure 4:**
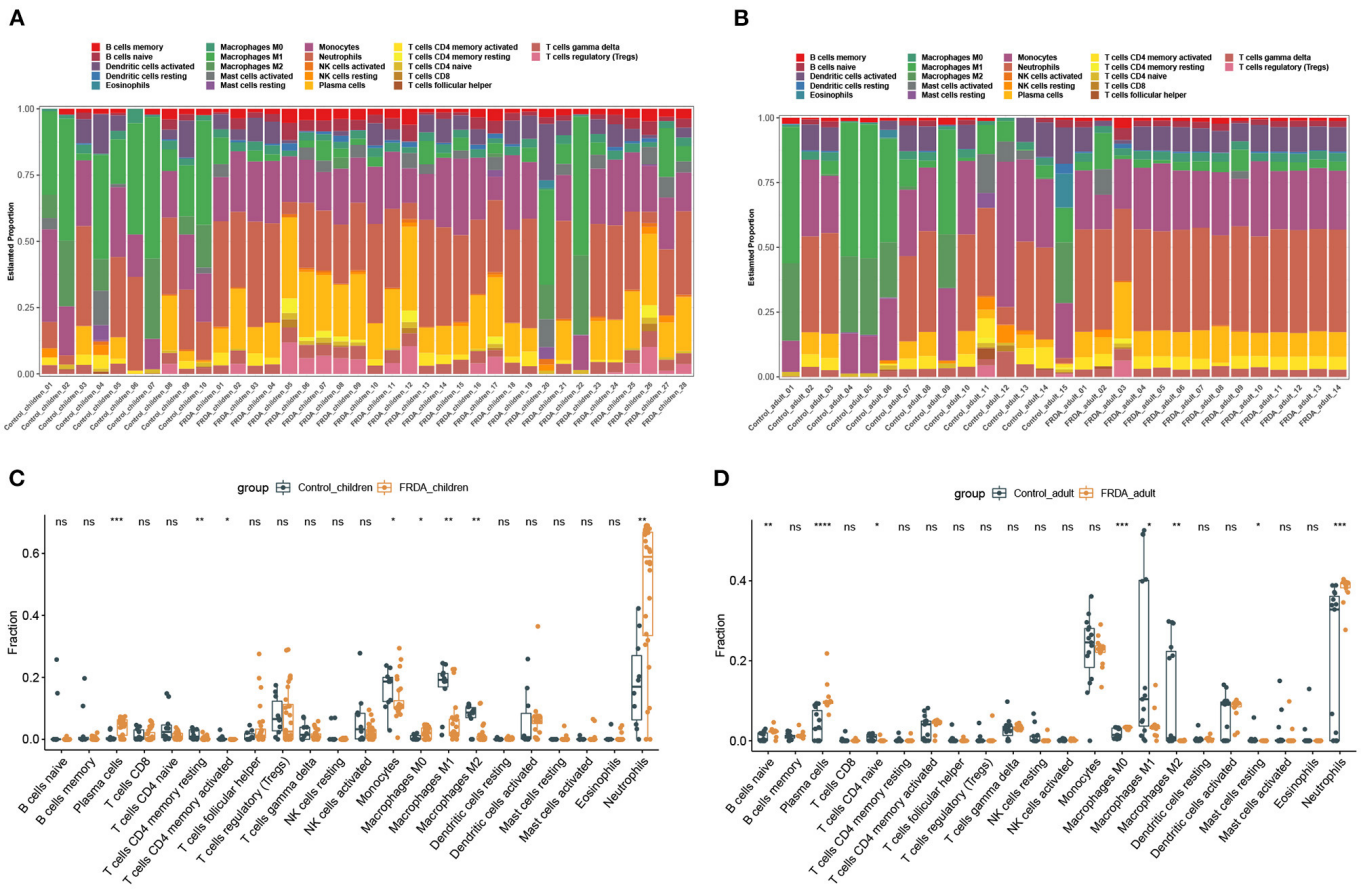
Immune infiltration analysis of co-DEGs in children and adult samples. **(A,B)** The landscape of all 22 types of immune cell components in children **(A)** and adults **(B)**. **(C,D)** Boxplots of the differentially infiltrated immune cells between the Control and FRDA group in children **(C)** and adults **(D)**. *p < 0.05, **p < 0.01, ***p < 0.001, ****p < 0.0001 when compared with the Control group.

Next, we perform the immune infiltration analysis of co-DEGs in child and adult datasets, respectively. The obvious proportions of immune cell subtypes were exhibited in different groups (Figures 4A,B). The number of Plasma cells, M0 Macrophages, and Neutrophils were significantly increased, while the number of resting memory CD4+ T cells, activated memory CD4+ T cells, Monocytes, M1 Macrophages, and M2 Macrophages were remarkably attenuated in the FRDA _children group (Figure 4C). Additionally, the FRDA _adult group notably elevated the number of naïve B cells, Plasma cells, M0 Macrophages, and Neutrophils, whereas markedly decreased the number of naïve T cells CD4, M1 Macrophages, M2 Macrophages, and resting Mast cells when compared with the Control_adult group (Figure 4D).

### PPI Network and Cluster Modules Analysis, Hub Genes Identification

**Figure 5:**
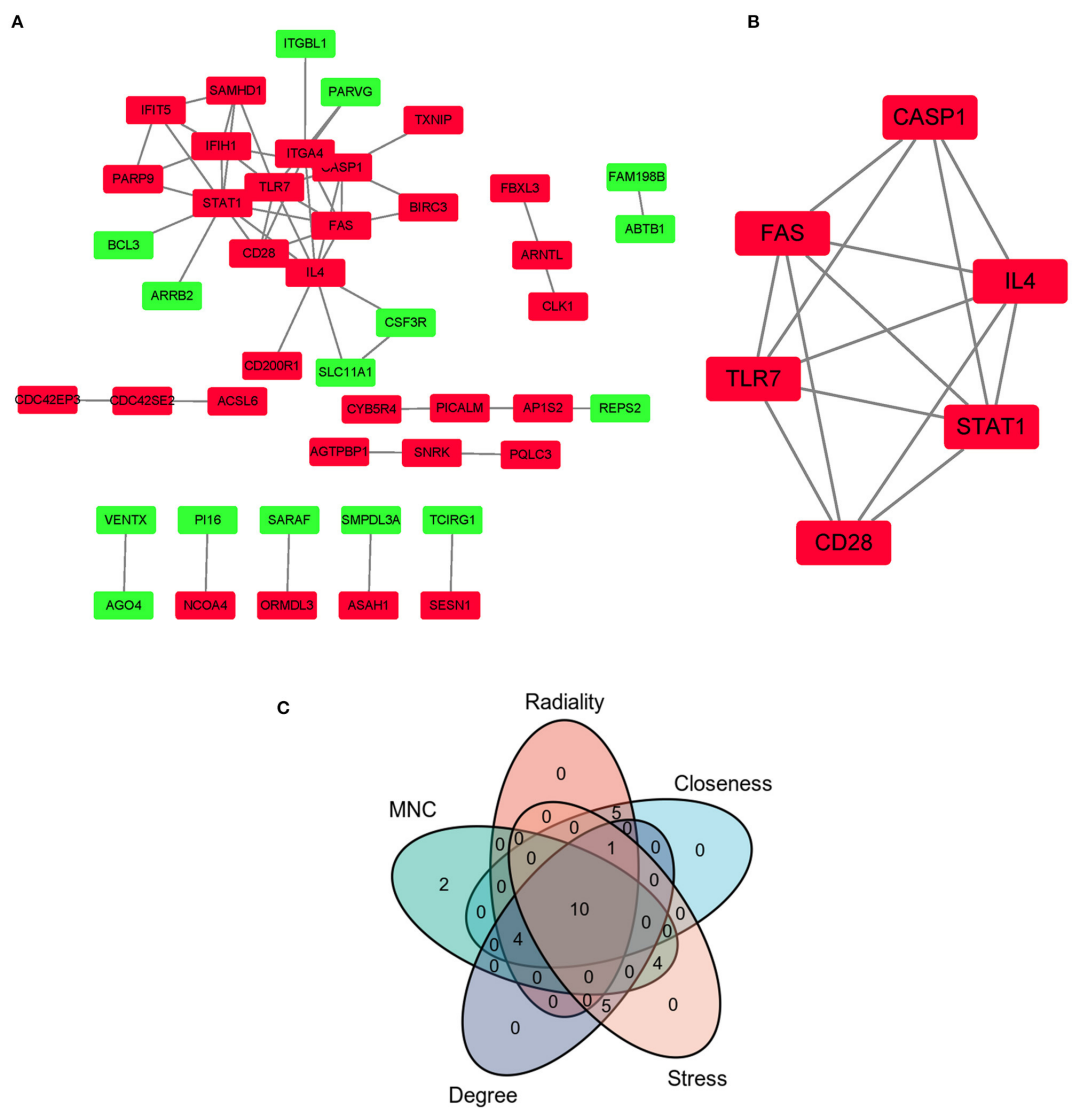
PPI network of co-DEGs and hub gene selection. **(A)** PPI network of co-DEGs was comprised of 45 nodes and 56 edges. Red rectangles: down-regulated genes; Green rectangles: up-regulated genes. **(B)** The key cluster module identified was by the MCODE plugin. **(C)** Hub genes were screened out by intersecting the first 20 genes in the five algorithms of cytoHubba.

The PPI network of co-DEGs, including 45 nodes and 56 edges, was constructed using the STRING website and visualized by Cytoscape software (Figure 5A). Next, we screened out a cluster module containing 6 down-regulated genes using the MCODE plugin (Figure 5B). Subsequently, a total of 10 hub genes were identified by intersecting the results of five algorithms from the cytoHubba plugin (Degree, MNC, Closeness, Stress, and Radiality) (Figure 5C). These identified hub genes were all significantly down-regulated in FRDA samples and were primarily enriched in the immune system process and response to other organisms (Table 2). These results evidence the major role of the declined expression of these hub genes in the pathogenesis of FRDA.

**Table 2:** A total of 10 hub genes were identified by intersecting the results of five algorithms from the cytoHubba plugin.

**Gene**	**log2FC**		***p*** **-value**		**Regulation**
	**Children**	**Adult**	**Children**	**Adult**	
**Immune system process**					
IFIH1	−1.33255	−2.19049	0.00744	0.00090	down
STAT1	−1.23078	−2.652	0.002283	2.26E-05	down
IL4	−1.63090	−1.09967	0.00020	0.03174	down
IFIT5	−1.51727	−1.40953	0.01086	0.01804	down
TLR7	−1.91344	−3.00512	0.00574	0.00090	down
FAS	−1.32193	−1.12326	0.00177	0.00011	down
CD28	−1.09838	−1.89092	0.02392	0.00018	down
ITGA4	−2.11557	−2.41976	0.00013	0.00000	down
SAMHD1	−3.10336	−1.40975	0.00011	0.01618	down
**Response to other organisms**					
CASP1	−2.82706	−3.38080	0.02651	0.00458	down

### Target miRNAs Mining, Construction of the Interaction Network, and Functional Enrichment Analysis of Target miRNAs

**Figure 6:**
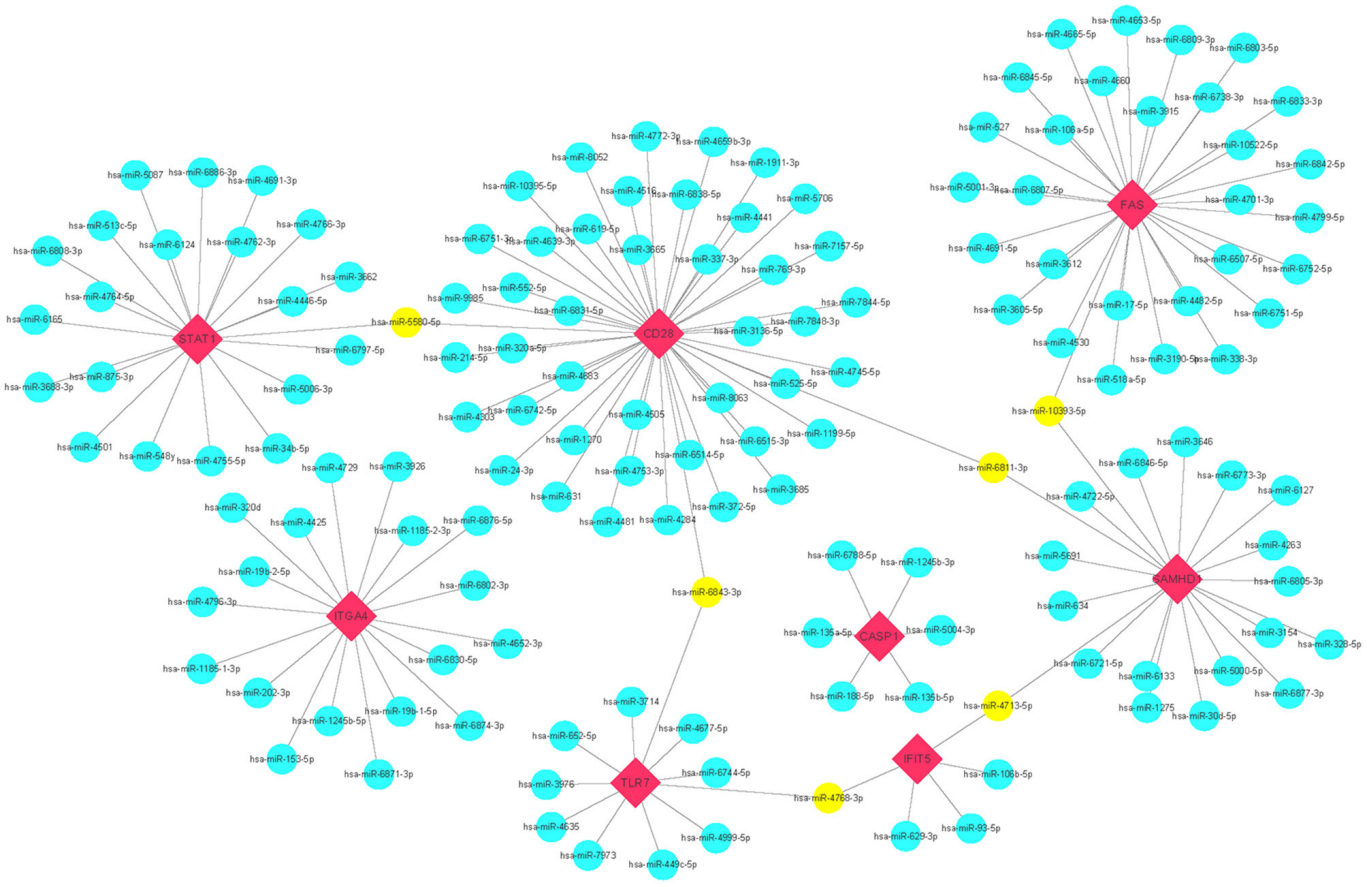
Predicted interactions among 8 hub genes and their target miRNAs. mRNA-miRNA co-expressed network was comprised of 158 nodes and 156 edges. Red diamonds: hub genes; Cyan circles: miRNAs; Yellow circles: miRNAs with more than 2 cross-linked genes.

miRNAs play a vital role in inducing gene degradation by binding the 3'UTR of mRNAs, thus exerting a negative regulation mechanism. We obtained a total of 150 target miRNAs of 8 identified hub genes and ascertained 156 mRNA-miRNA pairs. In addition, based on the prediction results, a mRNA-miRNA interaction network with 158 nodes and 156 edges was constructed and visualized by the Cytoscape software (Figure 6). The miRNAs with a greater number of cross-linked genes (≥2) were identified (Table 3).

**Table 3:** Predicted miRNAs and genes targeted by miRNAs.

**miRNA**	**Genes targeted by miRNA**	**Gene count**
hsa-miR-6843-3p	TLR7, CD28	2
hsa-miR-6811-3p	SAMHD1, CD28	2
hsa-miR-5580-5p	STAT1, CD28	2
hsa-miR-4768-3p	TLR7, IFIT5	2
hsa-miR-4713-5p	SAMHD1, IFIT5	2
hsa-miR-10393-5p	SAMHD1, FAS	2

**Figure 7:**
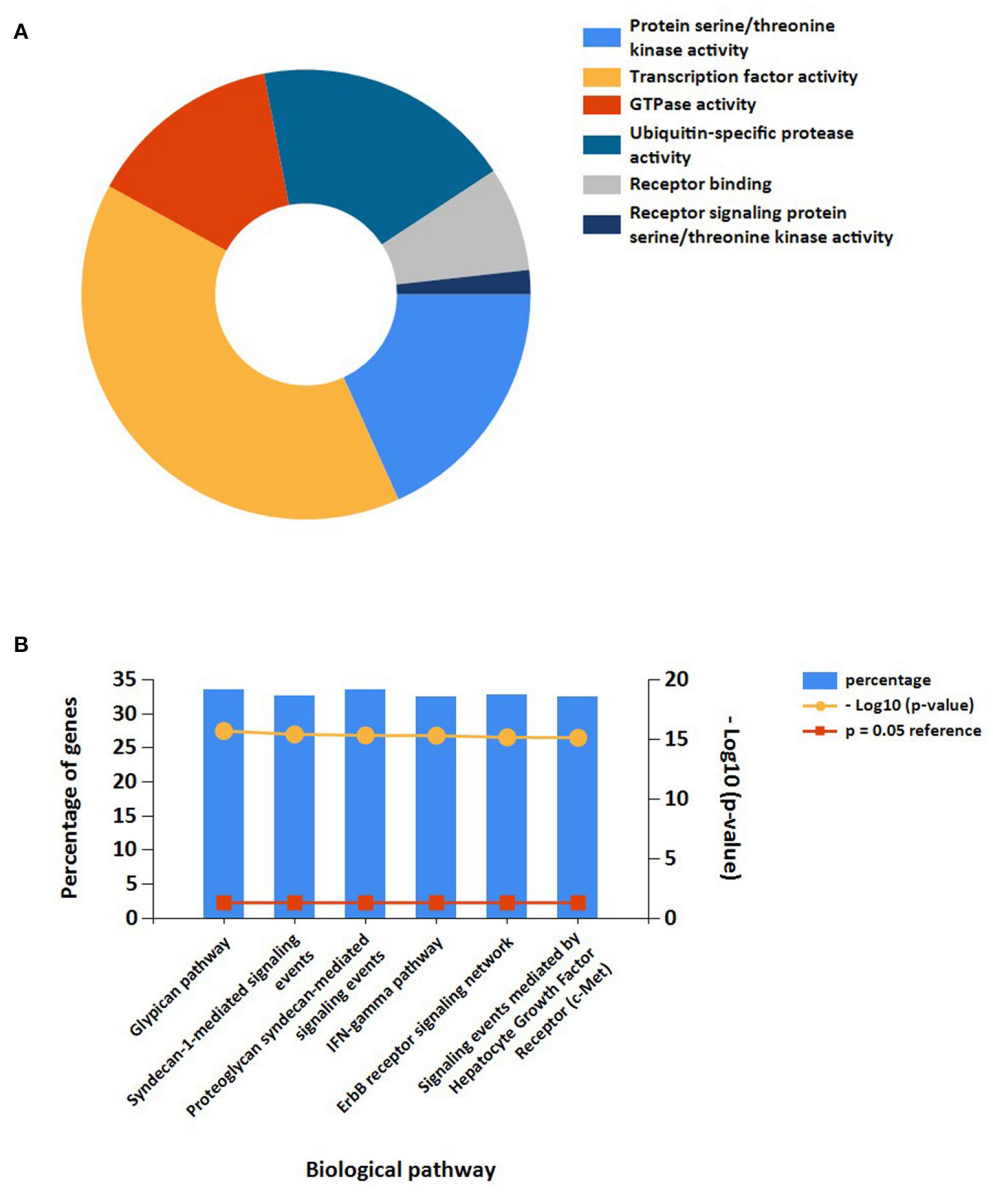
Functional enrichment analysis of target miRNAs. **(A)** Representative Pie chart of molecular functions of target miRNAs. **(B)** Representative Bar chart of Biological pathways of target miRNAs.

In addition, the results of miRNAs functional analysis indicated that the molecular functions were markedly enriched in protein serine/threonine kinase activity, transcription factor activity, GTPase activity, ubiquitin-specific protease activity, receptor binding, and receptor signaling protein serine/threonine kinase activity. The main biological pathways involved were glypican pathway, syndecan-1-mediated signaling, proteoglycan syndecan-mediated signaling, IFN-gamma pathway, ErbB receptor signaling, and c-Met-mediated signaling (Figures 7A,B).

### Validation of the 8 Hub Genes Expression in the GSE30933 Dataset

**Figure 8:**
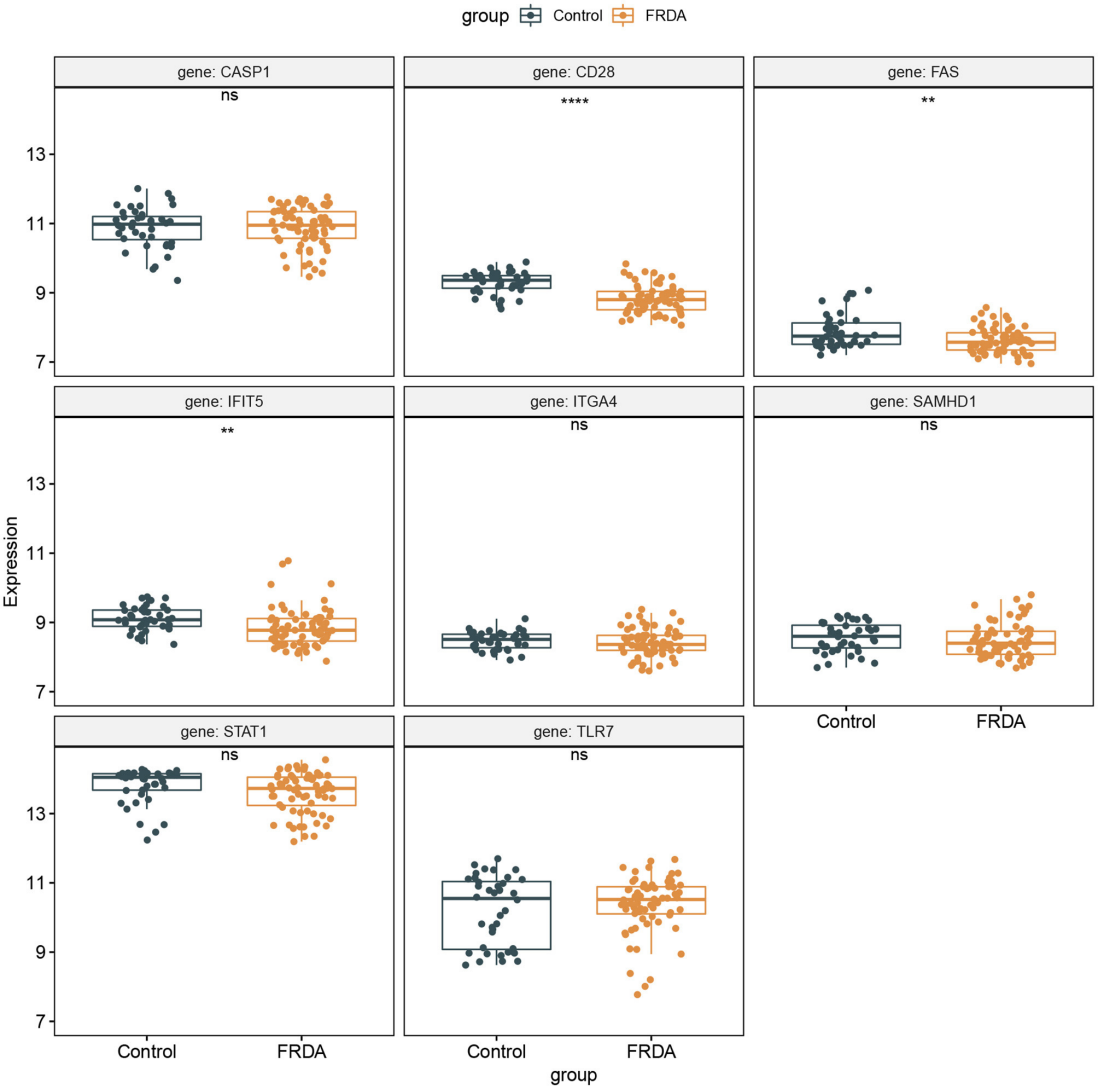
Validation of the 8 specifically expressed core genes using the GSE30933 dataset. **p < 0.01, ****p < 0.0001 when compared with the Control group.

**Figure 9:**
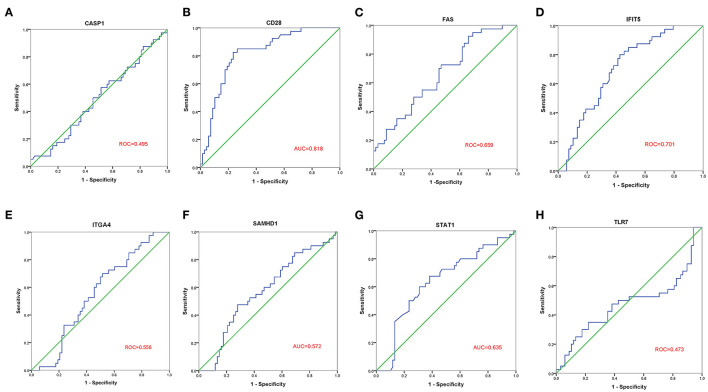
ROC curve analysis of the 8 specifically expressed core genes using the GSE30933 dataset. **(A–H)** ROC curve analysis of the 8 specifically expressed core genes.

The GSE30933 dataset, which included 40 healthy and 68 FRDA samples, was applied for validating the expression of 8 hub genes that interacted with target miRNAs. We found the mRNA expression of CD28, FAS, and IFIT5 were significantly attenuated in the FRDA group when compared with the Control group (Figure 8). Next, we performed SPSS software to analyze the expression profiles of 8 hub genes in healthy and FRDA samples and draw the ROC curves. CD28 (AUC = 0.818), FAS (AUC = 0.659), and IFIT5 (AUC = 0.701) genes all had the ability to differentiate FRDA from normal samples, and CD28 had the highest diagnostic value in FRDA samples (Figures 9A–H). Therefore, combined with the expression levels of these hub genes in the GSE30933 dataset, we assume that the downregulation of CD28, FAS, and IFIT5 might be potential diagnostic biomarkers for FRDA progression.

### Target lncRNAs Prediction and Construction of ceRNA Networks

**Figure 10:**
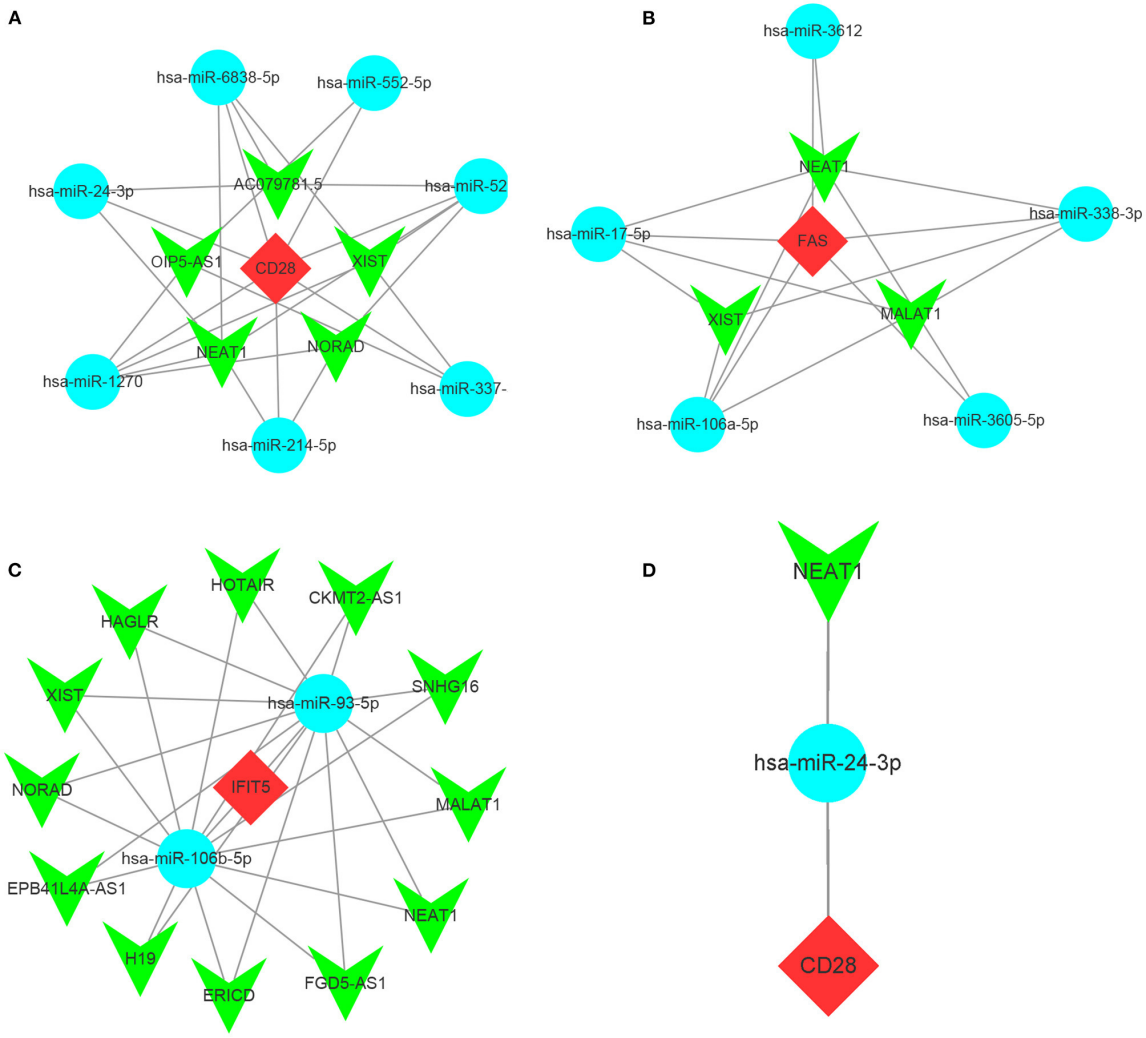
Construction of ceRNA networks and the identification of potential RNA regulatory pathways (a-c) Construction of ceRNA network of CD25 **(A)**, FAS **(B)**, and IFIT5 **(C)**. **(D)** NEAT1-hsa-miR-24-3p-CD28 RNA regulatory pathway. Red diamonds: hub genes; Cyan circles: miRNAs; Green V: lncRNAs.

As the upstream molecules of miRNAs, lncRNAs could regulate the biological function of miRNAs. Therefore, we predicted the target lncRNAs of the miRNAs interacting with CD28, FAS, and IFIT5 genes. A total of 5 target lncRNAs were obtained in the CD28-miRNA interaction network, 3 and 12 target lncRNAs were identified in FAS-miRNA and IFIT5-miRNA interaction networks, respectively. Three ceRNA networks were established and visualized by Cytoscape software (Figures 10A–C). Subsequently, we performed a literature search and only found miR-24-3p had been reported in FRDA. Therefore, we proposed that NEAT1-hsa-miR-24-3p-CD28 may be the potential RNA regulatory pathway involved in the progression of child and adult FRDA (Figure 10D).

## Discussion

FRDA is an autosomal recessive genetic disease with multiple system damage. In recent years, bioinformatics analysis has been widely developed and applied in various diseases, which reveals the underlying pathogenesis of disease and identifies vital biomarkers related to the diagnosis and prognosis of the disease (26). Nevertheless, the comprehensive research on the relationship between child and adult FRDA based on bioinformatics has not been systematically reported so far.

In this study, we screened out 530 DEGs in child samples, including 177 up-regulated and 353 down-regulated genes, and 857 DEGs in adult samples, including 483 up-regulated and 374 down-regulated genes. Eventually, 88 co-DEGs were identified by intersecting the up-regulated and down-regulated DEGs between the child and adult datasets. The results of GSEA and immune infiltration analysis indicated that these genes in both the child and adult datasets were mainly enriched in the immune response. GO and KEGG pathway enrichment analysis of co-DEGs suggested that the immune response characterized by the activation of immune cells and regulation of innate immune response were significantly stronger in FRDA samples. In addition, Reactome analysis revealed that immune system activation, necrosis, and signal transduction were closely related to the progression of child and adult FRDA.

Dysfunction of the immune system function is vital for the prognosis of diseases, including FRDA. Recently, Nachun et al. demonstrate that there are significant differences in the proportion of natural killer (NK) cells among control, carrier, and FRDA groups through bioinformatics analysis, and they are found significantly decreased in FRDA patients (27). In addition, IL-6, a cytokine produced by macrophages, has been proven to be increased in the blood plasma of FRDA patients, which suggests the activation of macrophages may be implicated in the neuropathology of FRDA (28). The current study, however, found a significant decrease in the number of macrophages and a remarkable increase in the number of activated NK cells in the FRDA_adult group; no statistical significance was found in the number of natural killer cells and macrophages between the FRDA_children and Control_children group. The following facts may have led to this discrepancy: Firstly, different datasets used for analysis create batch effects, thus may result in distinct results. Secondly, subjects from different regions or with different ethnicities may also have a certain impact on the results. Thirdly, in our current study, we have classified FRDA into adult and child groups and explored the association between FRDA and immune cell types in adults and children, which may also be another factor for the inconsistent results. Moreover, in our study, we performed the CIBERSORT algorithm instead of the quadratic programming method for immune infiltration analysis, which suggests that the impact of distinct analysis methods on the results could also not be ignored.

In order to further narrow the scope of research, we constructed the PPI network of co-DEGs, and screened out a total of 10 hub genes by intersecting the results of five algorithms in the CytoHubba plugin. These genes were mainly involved in the immune system process and response to other organisms. Afterward, we selected eight hub genes that interact with target miRNA and verified these hub genes using the GSE30933 dataset. We found the expression of three immune-related genes (CD28, FAS, and IFIT5) in FRDA samples were significantly lower than that in the Control group. The ROC analysis revealed these genes had greater diagnostic significance for FRDA. Therefore, we hypothesize that the downregulation of CD28, FAS, and IFIT5 may be the potential mechanisms involved in the progression of FRDA.

CD28, a member of cell surface glycoprotein receptor, primarily expressed on CD^4+^ T cells and CD^8+^ T cells, belongs to costimulatory molecules superfamily and plays a vital role in immune system response including T cell proliferation and differentiation, the production of cytokine and chemokines (28, 29). However, CD28 has not been mentioned in FRDA-related studies. In our current study, we found CD28 was remarkably down-regulated in both child and adult FRDA. In addition, the results of GSEA indicated that the T cell receptor signaling pathway was negatively correlated with child FRDA, and the ability of T cell differentiation was markedly inhibited in adult FRDA. These results revealed the insult of FRDA to the T cells-related immune process to some extent. Therefore, we conclude the downregulation of CD28 might play a critical role in the progression of children and adult FRDA.

FAS (also known as CD95 and TNFRSF6), a death receptor, belongs to the tumor necrosis factor (TNF) receptor superfamily and is mainly involved in the regulation of caspase-8-dependent apoptosis by interacting with its ligand FasL (30). Several studies have found the expression of FAS in plasma, gray matter, and white matter is significantly enhanced in Alzheimer's disease (AD) patients (31–33). This indicates that FAS might be markedly related to the progression of AD. However, recent studies also demonstrate that FAS engagement evokes non-apoptotic signals including cell migration and differentiation and cytokine processing (34, 35). Consistently, our study found plasma FAS decreased in both child and adult FRDA samples. Combined with the result of ROC analysis, we considered FAS as a potent protective factor for FRDA, and the downregulation of FAS may be closely related to the progression of FRDA in children and adults.

IFIT5, a member of the IFIT1 family, can be activated under stress conditions including virus infection, the production of type I interferon, and lipopolysaccharides stimulation (36, 37). IFIT5 has been proven to be implicated in the regulation of a wide variety of functions, such as viral restriction, translation initiation, cell migration and proliferation, and double-stranded RNA signaling (38, 39). Currently, IFIT5 has not been reported in FRDA. In our study, we identified that IFIT5 has a low level of expression in both child and adult FRDA, which indicated that the decrease of IFIT5 may be a key factor leading to pathological changes in FRDA.

Moreover, in order to clarify the potential regulatory mechanisms related to FRDA progression at the transcriptome level, we predicted the target lncRNAs of the miRNAs interacting with CD28, FAS, and IFIT5 genes and constructed a ceRNA network using Cytoscape software. Afterward, we applied the literature search and only found miR-24-3p was linked with ataxia (40). Therefore, we propose that NEAT1-hsa-miR-24-3p-CD28 may be the potential RNA regulatory pathway involved in the progression of child and adult FRDA.

Several limitations need to be highlighted in this study. Firstly, the sample size for analysis and validation is relatively insufficient. A greater number of samples are needed to verify these results. Secondly, our study preliminarily identified the potential RNA regulatory pathways during the progression of child and adult FRDA, which needs to be further clarified *in vitro, in vivo*, and clinical trials studies.

## Conclusion

In summary, our study found the downregulation of three immune-specific hub genes, CD28, FAS, and IFIT5, may be associated with the progression of child and adult FRDA. Furthermore, NEAT1-hsa-miR-24-3p-CD28 may be a potential RNA regulatory pathway related to the pathogenesis of child and adult FRDA. These findings provide a novel perspective for exploring the pathophysiological mechanism of FRDA progression at the transcriptome level.

## Data Availability Statement

Publicly available datasets were analyzed in this study. This data can be found here: National Center for Biotechnology Information (NCBI) Gene Expression Omnibus (GEO), https://www.ncbi.nlm.nih.gov/geo/, GSE11204 and GSE30933.

## Author Contributions

LL and YL designed the study. ZZ and QF collected and analyzed the data and searched the literature. YL and ZZ interpreted the results. LL wrote and prepared the original manuscript. YL revised the manuscript. All authors have read and approved the final manuscript.

## Conflict of Interest

The authors declare that the research was conducted in the absence of any commercial or financial relationships that could be construed as a potential conflict of interest.

## Publisher's Note

All claims expressed in this article are solely those of the authors and do not necessarily represent those of their affiliated organizations, or those of the publisher, the editors and the reviewers. Any product that may be evaluated in this article, or claim that may be made by its manufacturer, is not guaranteed or endorsed by the publisher.

## Abbreviations

FRDA: Friedreich's ataxia
GEO: Gene Expression Omnibus
DEGs: Differentially expressed genes
FDR: false detection rate
co-DEGs: co-expressed Differentially expressed genes
PPI: Protein-protein interaction
GO: Gene Ontology
KEGG: Kyoto Encyclopedia of Genes and Genomes
ceRNA: Competitive endogenous RNA
GSEA: Gene Set Enrichment Analysis
RMA: Robust Multiarray Average
lncRNAs: long noncoding RNAs
FC: fold-changes
NES: normalized enrichment score
DAVID: Database for Annotation, Visualization, and Integrated Discovery
BP: biological process
CC: cell composition
MF: molecular function
MCODE: Minimal Common Oncology Data Elements
MNC: Maximum Neighborhood Component
ROC: Receiver operating characteristic
AUC: Area under the ROC curve.
