# Supplementary figures and images for: Downregulation of Three Immune-Specific Core Genes and the Regulatory Pathways in Children and Adult Friedreich's Ataxia: A Comprehensive Analysis Based on Microarray
Source: Front Neurol. 2022 Feb 14;12:816393. doi: 10.3389/fneur.2021.816393 (PMC8884172; doi:10.3389/fneur.2021.816393)

A

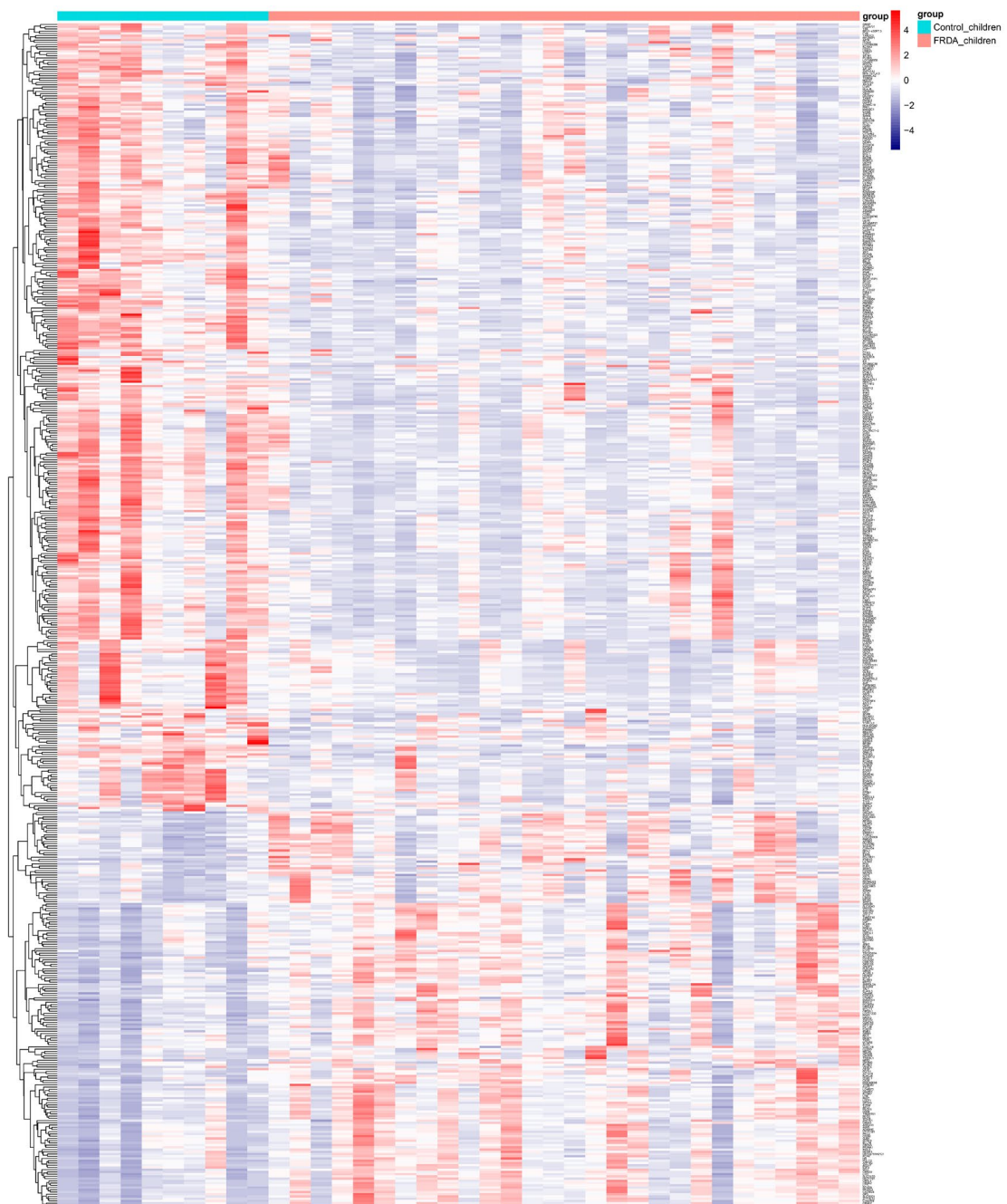

**B**

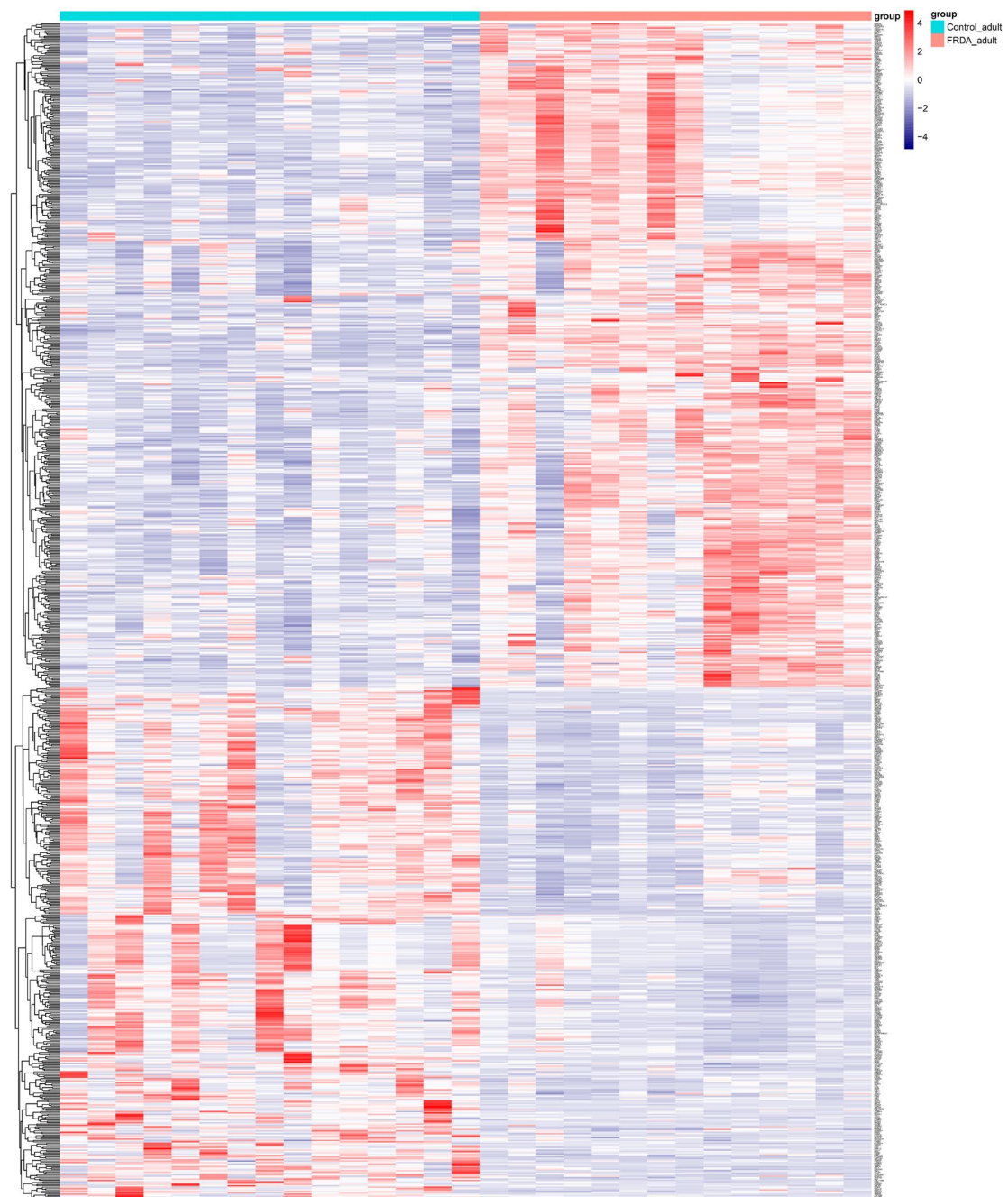

**Supplementary FIGURE 1** Representative heatmaps of all DEGs in children (A) and adult (B) samples.

Supplement: Supplementary file 2 [file Image_1.PDF]
